# Efficient Lithium Growth Control from Ordered Nitrogen‐Chelated Lithium‐Ion for High Performance Lithium Metal Batteries

**DOI:** 10.1002/advs.202002144

**Published:** 2020-11-19

**Authors:** Woo Hyeong Sim, Hyung Mo Jeong

**Affiliations:** ^1^ School of Mechanical Engineering Sungkyunkwan University 2066 Seobu‐ro Suwon 16419 Republic of Korea

**Keywords:** 3D lithium guiding matrix, bipyridinic sites, lithiophilic site, lithium growth control, uniform lithium ion flux

## Abstract

Lithium (Li) metal has attracted significant attention as next‐generation anode material owing to its high theoretical specific capacity and low potential. For enabling the practical application of Li‐metal as an anode according to energy demands, suppressing dendrite growth by controlling the Li‐ion (Li^+^) is crucial. In this study, metal–organic frameworks comprising bipyridinic nitrogen linker (M‐bpyN) are proposed as 3‐dimensional (3D) Li guiding matrix. The proposed approach creates ordered electronegative functional sites that enable the preoccupied Li^+^ in the ordered bipyridine sites to produce isotropic Li growth. The Li guiding matrix containing 3D ordered bipyridinic N sites introduces preoccupied Li^+^ sites that attract the Li growth direction, thereby suppressing the dendrite growth during the electrodeposition of Li. After applying the M‐bpyN layers, stable lifespan of up to 900 cycles in the Li|M‐bpyN|Cu cell and over 1500 h of operation in the Li|M‐bpyN|Li symmetric cell is achieved. Moreover, the Li|M‐bpyN|LiFePO_4_ configuration shows a long cycle retention of 350 cycles at 0.5 C. These results indicate that an M‐bpyN Li guiding matrix, which enables a uniform Li^+^ flux by 3D ordered Li^+^‐chelating sites, serve as a suitable host for Li^+^ and enhance the performance of Li‐metal electrodes.

## Graphical abstract

The lithium (Li) guiding matrix containing 3‐dimensional (3D) ordered bipyridinic nitrogen (N) sites introduces the uniform Li‐ion (Li^+^)flux from preoccupied Li^+^ sites attracting the Li growth direction, thus establishing the suppressed dendrite growth during the electrodeposition of Li, and the robust cycle operation of up to 900 cycle in half‐cell configuration and over 1500 h stable operation in symmetric‐cell configuration.

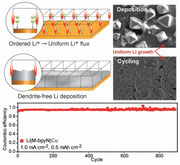

## 1 Introduction

With an increasing energy demand for electronic applications (e.g., electric vehicles and portable electronic devices), next‐generation batteries with high energy density have become essential.^[^ ^1^ ^]^ Lithium (Li)‐metal electrode has played a crucial role in realizing the next‐generation Li–oxygen, Li–sulfur, and Li‐metal batteries, which represent a new paradigm for practical applications. Li‐metal electrodes are considered as one of the most promising candidates for anode of next‐generation battery systems owing to the high theoretical specific capacity (3860 mAh g^−1^), low potential (−3.040 V vs standard hydrogen electrode), and low density (0.534 g cm^−3^) of Li‐metal.^[^ ^2^ ^]^ However, using Li‐metal electrodes can result in serious problems, such as internal shorts and explosion hazards, because Li metals tend to deposit in a dendritic form under electrochemical reduction.^[^ ^3^ ^]^ Uncontrolled dendritic Li growth associated with the irregular Li‐ion (Li^+^) concentration gradient from the unstable solid‐electrolyte interphase (SEI) layers leads to dead Li on the electrodes, thereby affecting the performance in repeated stripping/plating.^[^ ^4^ ^]^

**Figure 1:**
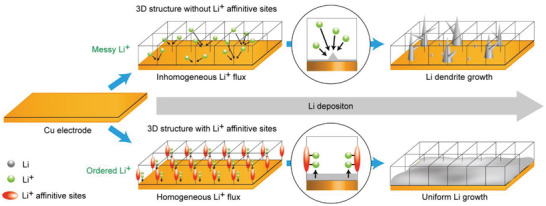
Schematic illustration of Li growth on a 3D matrix layer of Cu foil and 3D matrix layer with Li^+^affinitive sites.

To buffer the Li dendrite growth, nanomaterials with a 3‐dimensional (3D) porous structure have been intensively investigated for their potential use as electrodes^[^ ^5^ ^]^ and protective layers.^[^ ^6^ ^]^ This is because of their large surface area and porosity, which induce the evenly distributed electric field to reduce the effects of the dendrite and volume expansion of Li. The use of 3D porous electrodes, such as 3D hierarchical porous carbon,^[^ ^7^ ^]^ 3D porous copper (Cu),^[^ ^8^ ^]^ and 3D nickel foam,^[^ ^9^ ^]^ controls the volume changes in the Li anode after charging/discharging. Protective layers, such as metal–organic frameworks (MOFs),^[^ ^10^ ^]^ hollow carbon nanosphere layer,^[^ ^11^ ^]^ and Al_2_O_3_ protective layer,^[^ ^12^ ^]^ physically hinder Li dendrite growth and promote the charge transfer owing to their structure, resulting in an increased life of Li‐metal batteries. In spite of the authors’ success in improving the electrochemical performance and delaying the dendrite growth to an extent, some dendrite growth eventually appears after certain threshold capacities are met, suggesting that dendrite growth is not fundamentally suppressed.^[^ ^13^ ^]^ Even when a solid‐state electrolyte is used for physical protection, the dendrite growth of Li‐metal can be propagated through the protection layers to counter electrodes.^[^ ^14^ ^]^ A fundamental approach that involves the control of Li growth behavior to suppress the dendritic Li should be used to address the known problems with Li–metal electrodes. Materials with lithiophilic sites can suppress the Li dendrite during the electrodeposition of Li–metal on electrodes.^[^ ^15^ ^]^ The favorable Li^+^ binding affinity on lithiophilic sites, such as nitrogen (N) in pyridine and amine functional groups, causes them to serve as stable Li nucleation sites originating from the homogeneous Li^+^ flux near the electrodes.^[^ ^16^ ^]^ As N‐configuration plays a key role in achieving Li growth control, the 3D frameworks configuring ordered Li nucleation sites may allow for both Li growth control and volume change buffering during repeated charging/discharging on Li‐metal electrodes (**Figure** **1**).

In this study, we focused on the Li guiding matrix layers comprising MOFs for configuring the 3D ordered bipyridinic N sites (M‐bpyN layers)^[^ ^17^ ^]^ and offering 3D ordered Li^+^‐chelating sites. M‐bpyN should be used as the 3D host structure accommodating the ordered Li^+^ affinitive sites to equalize the Li growth rate on Li‐metal electrodes. Fortunately, as MOFs have a high surface area matrix with a periodic structure that comprise various metal clusters and organic linkers, a specific series of MOFs with unique structures can buffer the volume change in both the Li‐metal electrodes and ordered Li^+^ predeposition sites, enabling a uniform Li^+^ flux. A uniform Li^+^ flux can lead to stable SEI layer formation and uniform Li nucleation, thereby suppressing Li dendrite growth.^[^ ^18^ ^]^ Considering that the Li dendrite propagation through the MOFs layers without Li affinitive sites is eventually the same as the dendrite growth on Cu foil, the M‐bpyN layers containing the electronegative N sites are expected to enable the isotropic Li growth on uniform nucleation sites to form dendrite‐free dense Li layers by 3D ordered Li^+^‐chelating sites. Li nucleation sites can be arranged by the chelated Li^+^ on the 3D ordered bipyridine sites, leading to suppressed Li dendrite growth during operation. Regarding the pattern of isotropic Li growth without dendrites, the cell configurations with M‐bpyN layers exhibit a long cycle retention up to 900 cycles at 1 mA cm^−2^ and stability during long‐term operation at various operation rates (1–5 mA cm^−2^) and areal capacities (1–3 mAh cm^−2^).

## 2 Results and Discussion

### 2.1 Fabrication of the Li Guiding Matrix Layers

**Figure 2:**
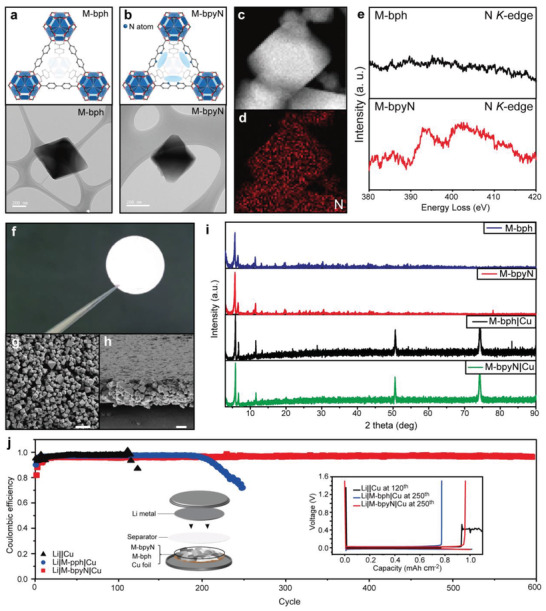
Fabrication of the 3D Li guiding matrixes and their electrochemical performances. a,b) Structure and TEM images of the as‐synthesized M‐bph and MbpyN. c) The STEM images of M‐bpyN. d) The EELS elemental mapping images of M‐bpyN (N: red). e) EELS spectra of M‐bph and M‐bpyN at N *K*‐edge. f) As‐synthesized M‐bpyN|Cu. g) Top‐view and h) cross‐section SEM images of M‐bpyN layers; SEM image scale bar of is 2 µm. i) X‐ray diffraction pattern of MOFs, M‐bph|Cu, and M‐bpyN|Cu. j) Cycle performances and voltage profiles of the Li||Cu, Li|M‐bph|Cu, and Li|M‐bpyN|Cu half‐cell configurations at a current density of 1.0 mA cm^−2^with an areal capacity of 1.0 mAh cm^−2^.

Generally, since MOFs comprise various organic linker and metal clusters, they have periodic porous structures and possess several characteristics.^[^ ^19^ ^]^ In addition, the properties of MOFs differ depending on the presence of functional sites in the organic linker. To compare the Li electrodeposition behavior for ordered Li^+^ predeposition sites in the MOF structure, zirconium (Zr) metal cluster‐based MOFs with biphenyl‐4,4′‐dicarboxylic acid (M‐bph) and 2,2′‐bipyridine‐5,5′dicarboxylic acid (M‐bpyN) were prepared (**Figure** **2**a,b). The average 500 nm crystal size and octahedral structures of the two types of MOFs were confirmed by transmission electron microscopy (TEM) analysis (Figure 2a,b), whereas M‐bpyN has previously been shown to contain N functional sites.^[^ ^17^ ^]^ Uniformly distributed N sites by bipyridine of M‐bpyN were observed by elemental mapping from electron energy loss spectroscopy (EELS) analysis (Figure 2c,d). N *K*‐edge peaks were shown in the energy loss spectra of M‐bpyN, while the characterized peak of energy loss by N was not detected at M‐bph (Figure 2e). When a different organic linker was used, M‐bpyN was found to have the same structure as M‐bph and contained bipyridine sites attracting the Li^+^. To fabricate the MOF layers for a Li‐metal half‐cell configuration, a mixture of M‐bpyN and binder was casted on Cu foils for M‐bpyN layers (Figure 2f). A slurry containing M‐bph and binder was also fabricated onto the layers consisting of M‐bph (M‐bph layers) to compare N effects. The morphology of the film comprised 5 µm thick M‐bpyN layers, as shown in scanning electron microscope (SEM) images of the top views and cross‐sections of the films with M‐bpyN layers (Figure 2g,h). In the continuous films, the overall shape of the as‐synthesized MOF particles is a polyhedron with an average diameter of ≈500 nm. Figure 2i shows the X‐ray diffraction (XRD) pattern of the MOF particles and M‐bpyN on a Cu foil (M‐bpyN|Cu) and M‐bph layers on a Cu foil (M‐bph|Cu). The main peaks seen below 2*θ* = 10° in the XRD pattern are attributable to the pores of the MOFs, and peaks are seen at 2*θ* = 5.7°.^[^ ^20^ ^]^ This indicates that the Zr cluster and organic ligand constitute an ordered structure with pores. In addition, the porous structure of the MOFs was also confirmed by the large surface area, which was obtained via Brunauer–Emmett–Teller analysis (Figure S1, Supporting Information). The structure and porous properties of the MOFs were maintained after the fabrication of MOF films on the Cu current collector for the half‐cell configuration. To check the electrochemical properties of the half‐cells with a Li guiding matrix, Li–Cu half‐cell configurations were assembled using M‐bph|Cu (for a Li|M‐bph|Cu cell) and M‐bpyN|Cu (for a Li|M‐bpyN|Cu cell) as the working electrodes and Li foil as the counter and reference electrodes, and then operated under specific current densities for repeated cycles (Figure 2j). The cycle retentions of the half‐cell configurations were evaluated under the areal capacity of 1.0 mAh cm^−2^ at a current density of 1.0  mA cm^−2^, as shown in Figure 2j. Li|M‐bpyN|Cu showed the most promising performance at the current density of 1.0 mA cm^−2^ with an areal capacity of 1.0 mAh cm^−2^, outperforming the cycle retention of half‐cells with Li||Cu (blank Cu foil) and Li|M‐bph|Cu. Furthermore, according to the voltage profiles (Figure 2j, inset), the blank cell showed a voltage fluctuation at the 120th cycle and server efficiency degradation that occurred around the 120th cycle. Relatively stable operation without voltage fluctuation was observed in Li|M‐bph|Cu, but substantial and irreversible degradation occurred in the 180th cycle. By contrast, the Li|M‐bpyN|Cu cell maintained high efficiency even after the 600th cycle without short circuit; this indicated that the M‐bpyN layers containing the ordered N‐configuration may be the essential component in suppressing the growth of Li dendrite during charging/discharging. In addition, the Li|M‐bpyN|Cu cell showed remarkably enhanced performance than other samples, even at a relatively low current density and areal capacity (Figure S2, Supporting Information). Electrochemical performances of Li|M‐bpyN|Cu along the thickness of M‐bpyN layers were also evaluated to check the effect of thickness of additional layers (Figure S3, Supporting Information). In this work, 5 µm thickness of M‐bpyN layers was selected for further electrochemical evaluation because the polarization increment and rapid degradation of life span were shown on the cells with thickened M‐bpyN layers (10 µm).

### 2.2 Effects of the Li Guiding Matrix Containing 3D Ordered Bipyridinic N Sites on Li Deposition Behavior

**Figure 3:**
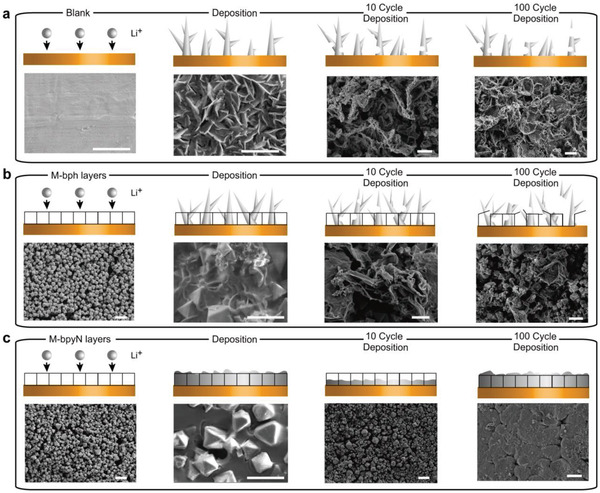
Li growth behavior on M‐bpyN|Cu. Schematic and SEM image of a) Cu foil, b) M‐bph|Cu, and c) M‐bpyN|Cu after Li deposition (4.0 mAh cm^−2^) and repeated charging–discharging at a current density of 1.0 mA cm^−2^with an areal capacity of 1.0 mAh cm^−2^. The scale bar for all SEM images is 2 µm.

To obtain evidence of the effects of the M‐bpyN layers on the half‐cell operation, the growth behavior of electrodeposited Li on the working electrodes was observed through SEM analysis (**Figure** **3**a–c). To compare the Li deposition behavior, a relatively high areal capacity of 4.0 mAh cm^−2^ was applied on the working electrodes with blank Cu foil, M‐bph|Cu, and M‐bpyN|Cu. On the blank Cu foil, Li dendrite growth was observed on the overall surfaces of the electrodes, which was attributed to the nonuniform electric fields on the Cu foil surface; this growth can be seen in schematic illustrations and SEM images (Figure 3a). On the M‐bph|Cu, although the dendrite growth can be partially retracted by the porous behavior of MOF particles, the continuous Li growth eventually induced the formation of Li dendrite over the M‐bph layers (Figure 3b). Considering the thickness (≈20 µm) of deposited Li at 1.0 mAh cm^−2^ (Figure S4, Supporting Information), such thin layer of 5 µm thickness is not sufficient to prevent the failure of cells without Li growth control. We confirm that the use of a porous protective layer without Li growth control will exclusively retard the appearance of Li dendrites on the electrodes by offering additional physical layers. In the case of M‐bpyN|Cu, SEM analysis shows the dendrite‐free surface of the electrode and densely grown Li layers before and after the excessive growth of Li on the electrode by discharging up to 4.0 mAh cm^−2^ (Figure 3c). As indicated by the Li growth behavior on M‐bpyN layers, the ordered pyridine sites in the M‐bpyN layers are expected to stimulate the regularly preoccupied Li^+^, thus allowing the stable and uniform Li^+^ flux to form an isotropic Li growth. From the viewpoint of long‐term behavior of each sample after the cycles, the deposited Li remained on the blank Cu foil and M‐bph layers due to the uncontrolled growth and repetitive destruction of dendrite. By contrast, the M‐bpyN layer can control the deposition of Li during charging/discharging, suggesting that deposited Li remains in a densely packed form as opposed to a dendritic form. The clear surface of M‐bpyN layer is also revealed after 100th Li stripping (Figure S5, Supporting Information). In addition, the formation of Li dendrite on electrodes can lead to the structural deformation of an MOF particle. To confirm the structural stability of the MOF particles during charging/discharging, TEM and energy dispersive X‐ray spectroscopy (EDS) analysis were performed on the MOF particle after repeated cycles (Figure S6, Supporting Information). The morphological structure of the MOF particle after cycling was observed through ex situ TEM because Zr‐based MOFs were applied in this study. The structure was distorted at M‐bph after repeated charging and discharging (Figure S6a–d, Supporting Information), because Li dendrites, through the MOF structure, were induced by the Li growth. Upon comparing the element mapping results, we found that the M‐bpyN retains its structure after 100 cycles (Figure S6e–h, Supporting Information). Moreover, after Li deposition, the evenly distributed Li element from deposited Li in the M‐bpyN particle were observed by the ex situ TEM (Figure S7, Supporting Information) and EELS elemental mapping analysis (Figure S8, Supporting Information), indicating that the uniform Li^+^ flux by Li^+^ affinitive sites of M‐bpyN induced the isotropic Li growth. These results support the idea that the dendritic Li was suppressed and grown as the dense Li layers within the M‐bpyN layers, which is in agreement with the ex situ SEM analysis results (Figure 3). Based on the electrochemical properties corresponding to the morphology analysis, the specificity of the ordered bipyridine sites in M‐bpyN layers can play a key role in controlling the Li growth.

**Figure 4:**
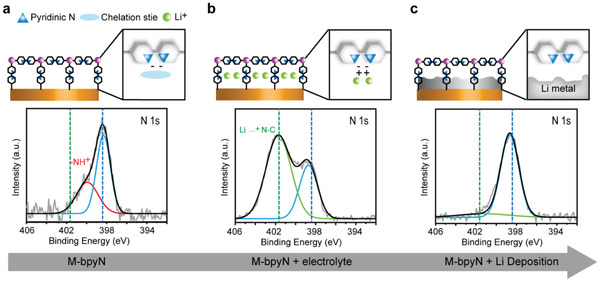
Chemical binding information of N1s in a) M‐bpyN layers, b) M‐bpyN layers after electrolyte soaking, and c) M‐bpyN layers after Li deposition.

As the Li growth behavior has been affected by the unique feature of the M‐bpyN layers, the chemical information of Li^+^ with pyridine sites has been analyzed using X‐ray photoelectron spectroscopy (XPS), and the results are shown in **Figure** **4**. To perform the XPS analysis, the samples were prepared with as‐synthesized M‐bpyN layers (Figure 4a), M‐bpyN layers after electrolyte soaking (Figure 4b), and M‐bpyN layers after Li electrodeposition (Figure 4c). In preparing the sample, 1 m lithium hexafluorophosphate (LiPF_6_) in ethylene carbonate (EC)/dimethyl carbonate (DMC) was chosen as the electrolyte because the spectra of N 1s in a lithium bis(trifluoromethanesulonyl)imide (LiTFSI)‐based electrolyte could overlap with information from the samples. Each sample underwent XPS analysis in N 1s to check the chemical state of N according to the behavior of Li^+^. The bipyridine sites in M‐bpyN layers are assigned to N 1s spectra at 398.4 eV, thus indicating pyridinic N sites (Figure 4a).^[^ ^21^ ^]^ After electrolyte soaking, the center of the N 1s spectra was split to higher binding energies because of the electron attraction by the cation resulting in Li—N binding;^[^ ^22^ ^]^ this indicated that the Li^+^ was chelated by the electronegative bipyridinic N and preoccupied in the ordered bipyridine sites of M‐bpyN layers (Figure 4b). During discharge, the preoccupied Li^+^ was expected to serve as the seed of Li‐metal growth. The peaks at high binding energies disappeared after discharge, suggesting that the Li—N binding was removed by the reduction of cations under Li growth (Figure 4c). Li^+^ chelating effects on the M‐bpyN layers were also confirmed by comparing the color change in the M‐bpyN layers, which can be seen in optical images of the M‐bpyN layers before and after Li contact (Figure S9a,b, Supporting Information). The color of the contact parts between the M‐bpyN layers and Li‐metal plate was changed to a dark color after undergoing activated conditions via heating and cycling. Li^+^ chelating of N sites in organic compounds darkens the color in a metal–organic complex.^[^ ^23^ ^]^ In addition, dark‐colored bipyridine linkers were also observed on the heated Li‐metal plate, thereby indicating the occurrence of Li^+^ chelating of pyridine sites (Figure S10, Supporting Information). The voltage profile of the initial Li nucleation of cells with M‐bpyN layers showed remarkably reduced nucleation overpotential (Figure S11, Supporting Information), which corresponded to the results of both XPS and SEM analyses. Notably, Li^+^ chelating on ordered bipyridine sites of M‐bpyN layers induced preoccupied Li^+^, which enabled the stable nucleation of Li‐metal and guided Li growth toward the densely packed Li growth layers.

### 2.3 Electrochemical Performances of Cell Configurations with the Li Guiding Matrix Containing 3D Ordered Bipyridinic N Sites

**Figure 5:**
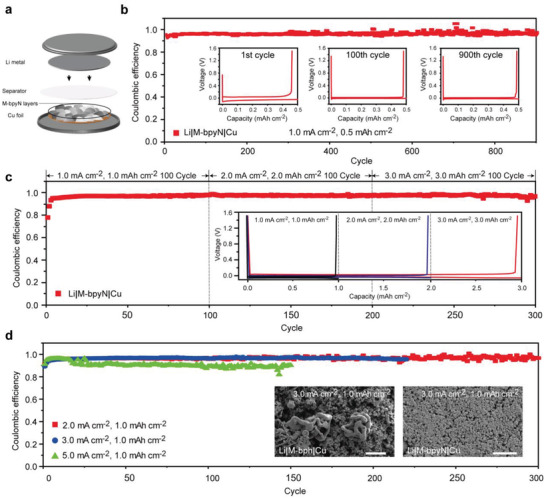
Electrochemical performances of the Li|M‐bpyN|Cu half‐cell configuration. a) Schematic of the Li|M‐bpyN|Cu configuration. b) CEs of Li|M‐bpyN|Cu at a current density of 1.0 mA cm^−2^, with an areal capacity of 0.5 mAh cm^−2^. c) Expanded properties of Li|M‐bpyN|Cu with various current densities ranging from 1.0 to 3.0 mA cm^−2^ for corresponding capacities from 1.0 to 3.0mAh cm^−2^. d) CEs and voltage profiles of Li|M‐bpyN|Cu half‐cells with an areal capacity of 1.0 mAh cm^−2^at current densities ranging from 2.0 to 5.0 mA cm^−2^. Surface morphology of M‐bph|Cu and M‐bpyN|Cu after 10 cycles. The SEM image shows the surfaces of M‐bph|Cu and M‐bpyN|Cu after being cycled at a current density of 3.0 mA cm^−2^. The scale bar is 2 µm.

To confirm the effects on the M‐bpyN layers with Li^+^ preoccupied sites, the Li|M‐bpyN|Cu configuration was further evaluated at various current densities and areal capacities (**Figure** **5**). The configuration scheme of Li|M‐bpyN|Cu is shown in Figure 5a. The M‐bpyN layers are located between the Li‐metal and Cu foil to control the behavior of Li^+^, thus allowing for stable operation. At a current density of 1.0 mA cm^−2^ with areal capacity of 0.5 mAh cm^−2^, the Li|M‐bpyN|Cu shows a high Coulombic efficiency (CE) of 98.4% during long‐term cycle operation up to 900 cycles (Figure 5b).The stable polarization voltage in the charge–discharge profile at each representative number of cycles corresponded to the outstanding cycle retention of the Li|M‐bpyN|Cu (Figure 5b, inset). To demonstrate the expandable properties of Li|M‐bpyN|Cu cells, current densities ranging from 1.0 to 3.0 mA cm^−2^ were applied on cells for corresponding capacities from 1.0 to 3.0 mAh cm^−2^ (Figure 5c). The cells were operated with stable CE (> 97%) during entire cycles under various current densities with corresponding capacities. Moreover, the stable operation under enlarged capacities of 3.0 mAh cm^−2^ allowed for the formation of a uniform Li^+^ flux along the 3D frameworks of M‐bpyN layers. The rate capability of the Li|M‐bpyN|Cu was also evaluated at various scan rates ranging from 2.0 to 5.0 mA cm^−2^ for areal capacity of 1.0 mAh cm^−2^ (Figure 5d). At the current density of 2.0 mA cm^−2^, the cycle retention was maintained over 300 cycles with stable charge–discharge profiles. Moreover, controlled Li growth on the M‐bpyN layers was observed with a high rate operation at 3.0 mA cm^−2^ (Figure 5d, inset), thereby supporting the excellent cycle retention of the Li|M‐bpyN|Cu. Even at high operation rates, the high CEs of 96.5% and 95.1% were maintained at current densities of 3.0 and 5.0 mA cm^−2^, respectively. By contrast, dendritic Li growth was observed on the M‐bph layers after the cycling operation at 3.0 mA cm^−2^, corresponding to the poor capacity retention of Li|M‐bph|Cu (Figure S12, Supporting Information). Based on the inset SEM image and CE data, the cycle retention at a relatively high operation rate was substantially improved by suppressing dendritic Li growth through the uniform Li^+^ flux by preoccupied Li^+^ on ordered bipyridine sites in M‐bpyN layers.

**Figure 6:**
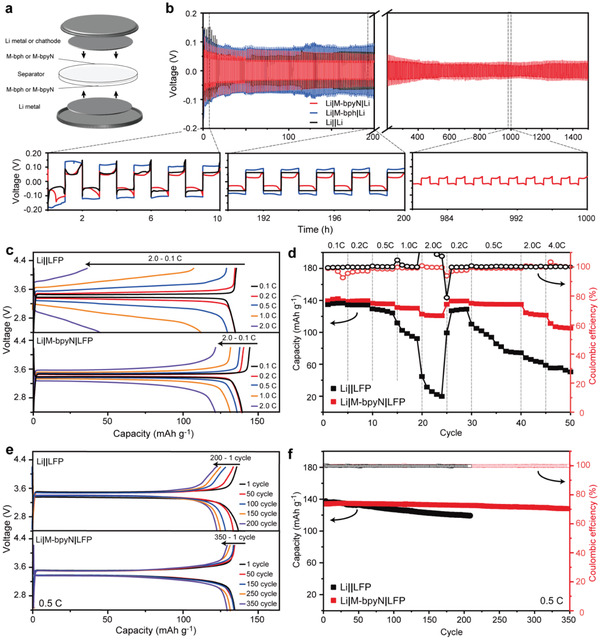
Electrochemical performances of symmetric (Li|M‐bpyN|Li) and asymmetric (Li|M‐bpyN|LFP) configurations. a) Schematic of cell configuration using M‐bpyN layers. b) Charge–discharge profile of Li||Li, Li|M‐bph|Li, and Li|M‐bpyN|Li for long‐term operation at a current density of 1.0 mA cm^−2^and areal capacity of 1.0 mAh cm^−2^. The specific range of profiles at 0–10, 40–50, and 980–1000 h were magnified to observe the charge–discharge behavior. c) Charge–discharge profiles and d) rate capabilities of Li|M‐bpyN|LFP and Li||LFP at various scan rates from 0.1 to 4.0 C. e) Charge–discharge profiles and f) capacity retention of Li|M‐bpyN|LFP and Li|LFP during repeated cycle at 0.5 C.

To evaluate the properties of the M‐bpyN layers in other configurations, Li symmetric cells were assembled and evaluated (**Figure** **6**a). These symmetric cells were configured to the two‐electrode system comprising two Li‐metal disks with a commercial separator (Li||Li), M‐bph layers (Li|M‐bph|Li), and M‐bpyN layers (Li|M‐bpyN|Li). The long‐term voltage hysteresis was obtained from the evaluation of symmetric‐cell configurations under the current density of 1.0 mA cm^−2^ for the areal capacity of 1.0 mAh cm^−2^ (Figure 6b). In the case of Li|M‐bpyN|Li, stable charge–discharge profiles were observed even after 1500 h with overpotential stabilization, indicating the formation of stable SEI layer are established by the Li growth control. Meanwhile, Li||Li and Li|M‐bph|Li show relatively high and increased overpotential during repeated charge–discharge. Due to uncontrolled Li growth in Li||Li and Li|M‐bph|Li, the SEI layer is constructed and destroyed continuously, resulting in induce higher surface resistance and overpotential.^[^ ^24^ ^]^ Moreover, symmetric configuration without Li growth control also shows a sudden potential drop without mass‐transfer limitation (no tails at the end of charging–discharging), indicating that the short circuit was caused by Li dendrite growth^[^ ^25^ ^]^ during cycle operation (Figure S13, Supporting Information). According to the voltage profiles in Figure S13 of the Supporting Information, a short circuit occurred on Li||Li and Li|M‐bph|Li as these configurations showed a trend similar to that in cells configured with Li metals without a separator (green solid line) to intentionally induce short circuit.^[^ ^25^ ^]^ Similar to the voltage profile trends, the overpotentials of Li||Li and Li|M‐bph|Li from potential hysteresis reached the sandwiched Li foils (Figure S14, Supporting Information). This result also corresponded to the dramatically decreased cell resistance observed by electrochemical impedance spectra (EIS) analysis (Figure S15, Supporting Information). The appearance of increased overpotential and short circuit in the symmetric configuration without Li growth control was supported by the dendritic Li growth on the Li|M‐bph|Li cells within 10 cycles (Figure S16a, Supporting Information). By contrast, the dendrite‐free morphology of M‐bpyN layers was observed in Li|M‐bpyN|Li, corresponding to the extremely stable operation of symmetric cells by uniform Li growth (Figure S16b, Supporting Information).

To evaluate the practical application of M‐bpyN layers toward Li‐metal electrodes, the cell configuration with a conventional cathode of LiFePO_4_ (Li|M‐bpyN|LFP) was assessed. The operation voltage window of the Li|M‐bpyN|LFP was obtained by combining the voltage profiles from the Li|M‐bpyN|Cu and Li||LFP half‐cell configurations to match the total charge of the cells (Figure S17a, Supporting Information). The discharge plateau of Li|M‐bpyN|LFP at the rate of 0.1 C appeared at 3.4 V and extended to the capacity of 140 mAh g^−1^ based on the mass of LFP contents (Figure S17b, Supporting Information), which corresponded to the representative voltages and capacities of the LFP electrodes.^[^ ^26^ ^]^ As the full‐cell configuration was optimized by combining the negative and positive electrodes, the charge–discharge profiles of Li|M‐bpyN|LFP showed stable operation without significant polarization at various scan rates ranging from 0.1 to 2 C (Figure 6c). In addition, the stable operation of Li|M‐bpyN|LFP resulted in comparable and reproducible performances throughout the overall cycle at various scan rates from 0.1 to 4 C (Figure 6d). The capacities at the relatively high scan rate of 2 C reached over 85% of the capacities at the rate of 0.1 C, outperforming the rate capability of Li||LFP cell configuration. The stable operation of Li|M‐bpyN|LFP also reserved the high capacity retention of over 96.2% at the rate of 0.5 C after 350 cycles, thus overcoming the problem related to poor capacity retention of Li||LFP (Figure 6e,f). The first CE of Li|M‐bpyN|LFP at the rate of 0.5 C was 99.9%, and this was retained during cycle performance at the rate of 0.5 C. Moreover, the stable cycle performance of Li|M‐bpyN|LFP with 1,3‐dioxolane (DOL)/1,2‐dimethoxyethane (DME) based electrolyte is consistent with that of cells using carbonate‐based electrolyte (Figure S18, Supporting Information). The unique feature of the M‐bpyN layers guiding the Li growth along the ordered nucleation sites can facilitate a high stability and rate capability in the Li|M‐bpyN|LFP configuration.

## 3 Conclusion

In this study, M‐bpyN was investigated as a 3D Li guiding matrix for high performance Li‐metal electrodes; the results showed that it promoted stable and uniform Li growth. The ordered bipyridine sites in M‐bpyN create Li^+^ affinitive sites, thereby guiding the isotopic Li‐metal growth owing to the uniform Li^+^ flux on the electrodes. Li^+^ chelating on bipyridine sites was demonstrated by XPS, corresponding to the reduced overpotential of Li stripping on M‐bpyN|Cu. The uniformly dense Li‐metal growth suppressing dendritic Li on electrodes led to high performances in multiple types of cell configurations using Li|M‐bpyN|Cu, Li|M‐bpyN|Li, and Li|M‐bpyN|LFP in various operating conditions. The Li|M‐bpyN|Cu configuration maintained stability over 900 repeated cycles. Relatively high current densities (1–5 mA cm^−2^) and areal capacities (1–3 mAh cm^−2^) on Cu foils were also stably operated under a long cycle retention. The Li|M‐bpyN|Li symmetric configuration exhibited a long cyclability of over 1500 h without any voltage drop, thereby suggesting its potential for practical application in commercially available LFP cathodes. The Li|M‐bpyN|LFP configuration maintained a high capacity retention of 96.2% over 350 cycles at 0.5 C with a stable charge–discharge operation. Therefore, we believe that the proposed method will provide a facile strategy for obtaining high performance Li‐metal electrodes via combined approaches of ordered Li^+^ flux in 3D guiding materials, ultimately enabling uniform Li growth.

## 4 Experimental Section

##### Materials

Zirconium chloride (ZrCl_4_, ≥99.5% trace metals basis), two types of linker (2,2′‐bipyridine‐5,5′‐dicarboxylic acid and biphenyl‐4,4′‐dicarboxylic acid, 97%), acetic acid (glacial, ReagentPlus ≥99%), trimethylamine (TEA, ≥99%), poly(vinylidene fluoride) (PVDF, average Mw = 534 000 by GPC, powder), 1‐methyl‐2‐pyrrolidinone (NMP, ReagentPlus, 99%), and electrolytes for all of the battery tests were purchased from Sigma‐Aldrich. Distilled water, *N*,*N*‐dimethylformamide (DMF, >98.8%), methanol (HPLC, 99.9%), and ethanol (HPLC, 99.9%) were purchased from Daejung Chemicals. Nafion D‐520 dispersion (5% w/w in water and 1‐propanol, >1.00 meq g^−1^ exchange capacity) was purchased from Alfa Aesar. The polypropylene (PP) separator (Celgard‐2400) was purchased from Celgard. Carbon black (Super P) and LiFePO_4_ (LFP) were purchased from MTI Korea. Lithium (Li) foil was purchased from Honjo Chemical Corp. Copper (Cu) foil and aluminum foil was purchased from UACJ Foil Corp.

##### Fabrication of 3D Li Guiding Matrix

To fabricate a 3D Li guiding matrix containing bipyridine and biphenyl, MOFs comprising bipyridine linker (M‐bpyN),^[^ ^27^ ^]^ and biphenyl linker (M‐bph)^[^ ^28^ ^]^ were used. M‐bpyN and M‐bph were synthesized using a common solvothermal method.^[^ ^27^, ^28^ ^]^ ZrCl_4_ powder and organic linker were each dispersed in 5 mL of DMF. Next, acetic acid (1.38 mL) was added into the solution of ZrCl_4_ and TEA (30 µL) was added into the solution of organic linkers. Then, the ZrCl_4_ and organic linker solutions were mixed and heated at 85 °C for 12 h. Following the reactions, the resultants were centrifuged and washed thrice with DMF and methanol. Finally, the MOF powder was dried overnight in a vacuum oven at 60 °C.

To fabricate the M‐bpyN and M‐bph layers, the MOF particles and Nafion binder were dispersed in ethanol at a mass ratio of 80:20 to form a slurry. Then, the slurry was casted onto a commercial PP separator and Cu current collector with an applicator. The coated layers were dried overnight in a vacuum oven at 60 °C. After drying, the Cu current collector and the PP separator were cut with diameters of 14 and 18 mm, respectively.

##### Fabrication of 3D Li Guiding Matrix—Characterization

Field emission SEM images were captured by SUPRA55VP (Carl Zeiss). Field emission TEM images and EDS mapping images were obtained using a JEM‐2100F (JEOL). ELS analysis was conducted with a Titan G2 TEM (FEI). For crystal structure analysis, XRD (X'pert PRO MPD, Malvern PANalytical) analysis was conducted. For each condition, XPS (K Alpha^+^, Thermo Scientific) analysis was performed to confirm the bonding state between pyridinic sites and Li ions in the electrolyte.

##### Fabrication of Cell Configuration

Li|M‐bph|Cu and Li|M‐bpyN|Cu half‐cells were assembled using a 2032‐type coin cell, with 14 mm diameter 3D matrix layers (M‐bph and M‐bpyN) on Cu or blank Cu foil current collector, and 12 mm diameter Li foil as the working and counter electrodes. The 70 µL of 1 m lithium bis(trifluoromethanesulonyl)imide (LiTFSI) in DOL/DME) solution with 2 wt% lithium nitrate (LiNO_3_) was used as the electrolyte for all batteries. The symmetric cells were assembled using 12 mm diameter Li‐metal disks for both electrodes. M‐bph or M‐bpyN coated PP was used as the separator. To evaluate the feasibility of the full‐cell configuration, an LFP electrode was prepared by casting a slurry of LFP, super P, and PVDF with NMP as the solvent in a 90:5:5 ratio. Then, the mixed slurry was coated onto aluminum foil and dried overnight in a vacuum oven at 60 °C. The full cell was assembled using a dried LFP electrode as cathode and Li‐foil as anode. The mass loading of LFP in cathode was determined to 10–15 mg cm^−2^ for full cell configuration. M‐bph or M‐bpyN coated PP was used as a separator for the full‐cells. The electrolyte of the full cell used 1 m LiPF_6_ ethylene carbonate/diethyl carbonate (1 m LiPF_6_ EC/DEC) with 5 wt% fluoroethylene carbonate and 1 m LiTFSI in DOL/DME with 2 wt% LiNO_3_. All coin cells were assembled in an Ar‐filled glove box. In the glove box, O_2_ and H_2_O were maintained below 0.1 ppm.

##### Electrochemical Measurement

All electrochemical battery tests were measured using a battery cycler (WBCS3000Le32, Won‐A‐Tech), and EIS analysis was conducted with a VMP3 (Bio‐logic). The half‐cells were precycled ten times at 0.02–1.0 V (vs Li^+^/Li) and 100 µA cm^−2^ for stable operation, then tested at various electrochemical conditions. The cutoff voltage of each half‐cell was fixed at 1.5 V. The charging and discharging current densities of the symmetric cell were both 1.0 mA cm^−2^ and the cut off capacity was 1.0 mAh cm^−2^. The voltage range of the Li||LFP full cell was set at 2.4–4.2 V.

## Conflict of Interest

The authors declare no conflict of interest.

## Supporting information

Supporting Information
