# Supplementary material for: Efficient Lithium Growth Control from Ordered Nitrogen‐Chelated Lithium‐Ion for High Performance Lithium Metal Batteries
Source: Adv Sci (Weinh). 2020 Nov 19;8(1):2002144. doi: 10.1002/advs.202002144 (PMC7788589; doi:10.1002/advs.202002144)
Supplement: Supplementary file 1 — Supporting Information [file ADVS-8-2002144-s001.pdf]

© 2020 Wiley-VCH GmbH

ADVANCED SCIENCE
Open Access

Supporting Information

for *Adv. Sci.*, DOI: 10.1002/advs.202002144

Efficient Lithium Growth Control from Ordered Nitrogen-chelated Lithium -ions for High Performance Lithium Metal Batteries

Woo Hyeong Sim and Hyung Mo Jeong *

Supporting Information

Efficient Lithium Growth Control from Ordered Nitrogen-chelated Lithium -ions for High Performance Lithium Metal Batteries

Woo Hyeong Sim and Hyung Mo Jeong *

W. H. Sim, Prof. H. M. Jeong
School of Mechanical Engineering, Sungkyunkwan University, 2066 Seobu-ro, Suwon, 16419, Republic of Korea
E-mail: hmjeong@skku.edu (Prof. Hyung Mo Jeong)

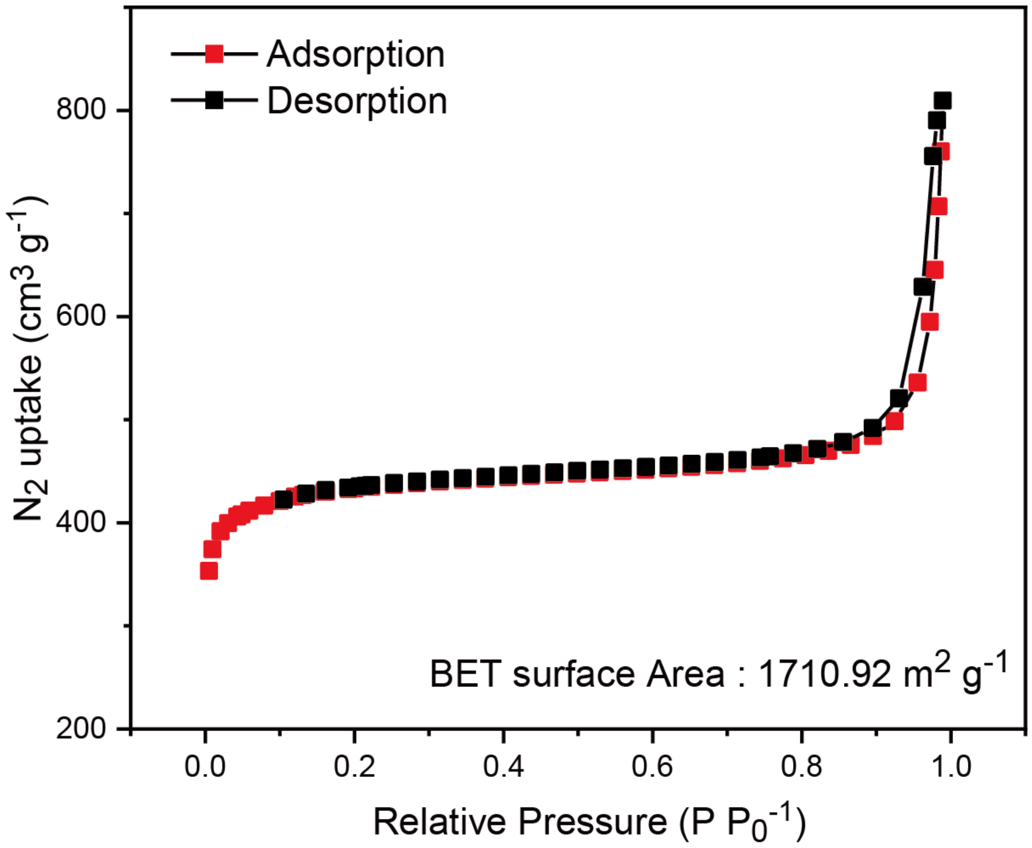

Figure S1. N_2 adsorption/desorption isotherm of M-bpyN powders.

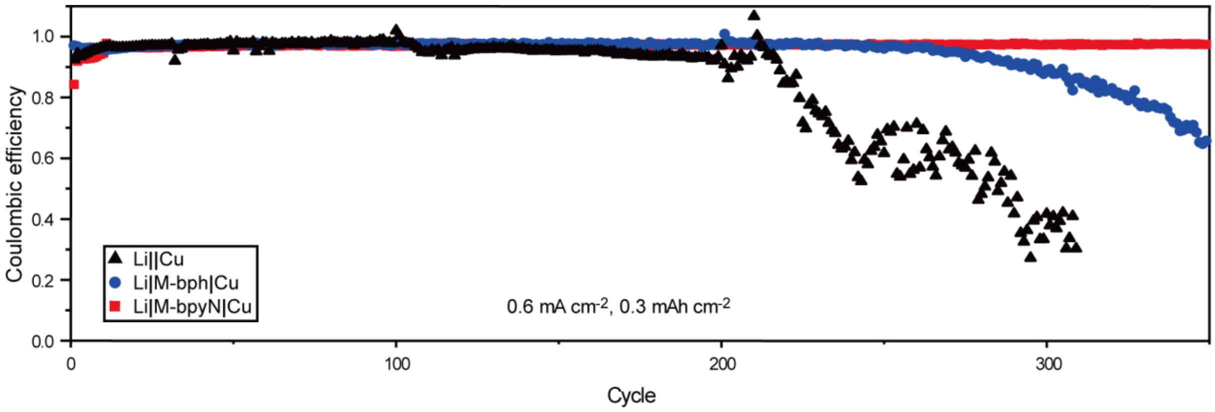

Figure S2. Cycle performances of Li||Cu, Li|M-bph|Cu, and Li|M-bpyN|Cu configurations at a current density of 0.6 mA cm^{-2} and areal capacity of 0.3 mAh cm^{-2}.

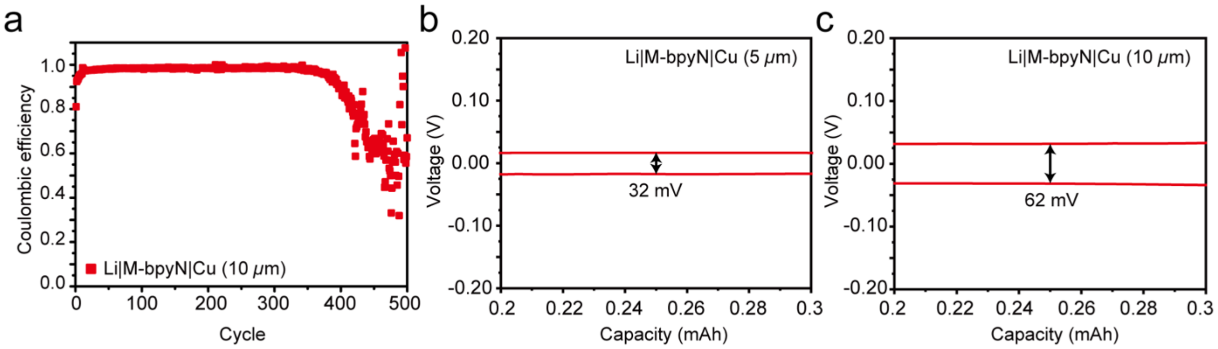

Figure S3. a) Cycle performance of Li|M-bph|Cu with increased thickness and mass loading of M-bpyN layers. b and c) the charge-discharge overpotentials of Li|M-bpyN|Cu (5 μm) and Li|M-bpyN|Cu (10 μm).

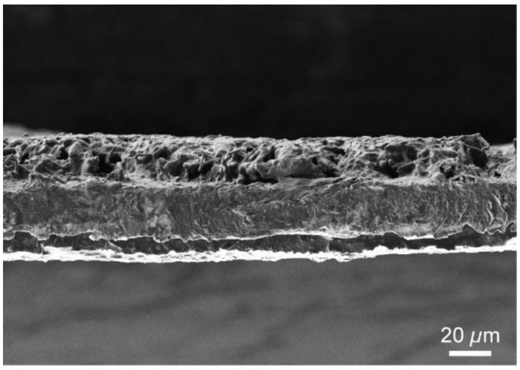

Figure S4. Cross-section SEM image of deposited Li after Li deposition (1.0 mAh cm^{-2}) on Cu foil.

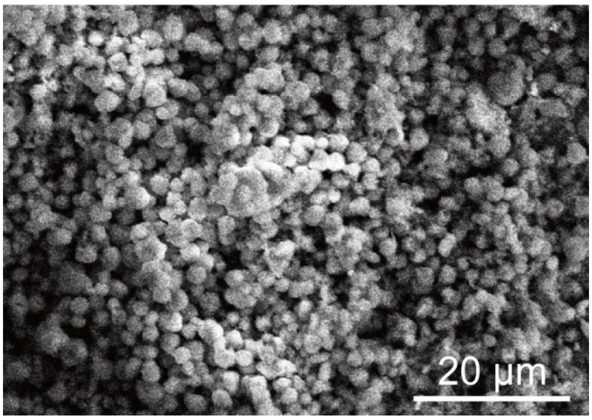

Figure S5. SEM image of M-bpyN|Cu after 100th stripping at a current density of 1.0 mA cm^{-2} with an areal capacity of 1.0 mAh cm^{-2}.

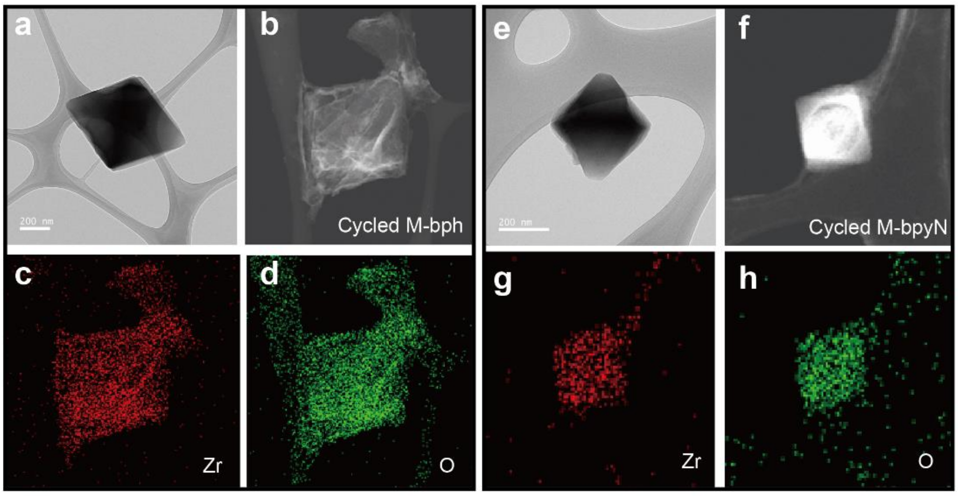

Figure S6. TEM analysis of a) M-bph and e) M-bpyN before cycling. TEM and EDS mapping of b-d) M-bph and f-h) M-bpyN after charging–discharging.

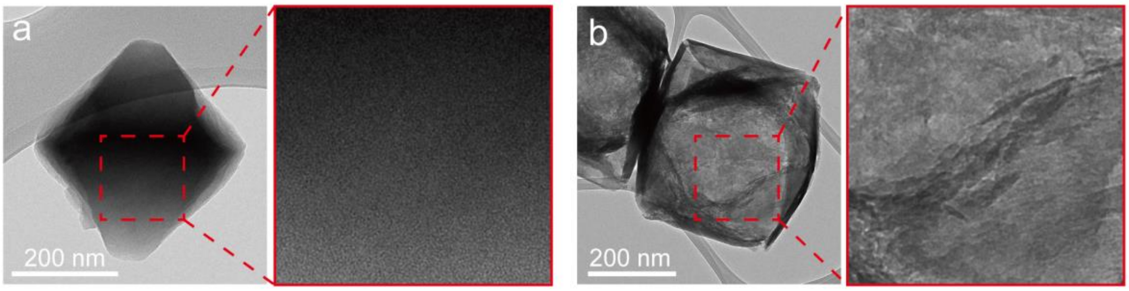

Figure S7. Ex-situ TEM image of M-bpyN before and after Li deposition

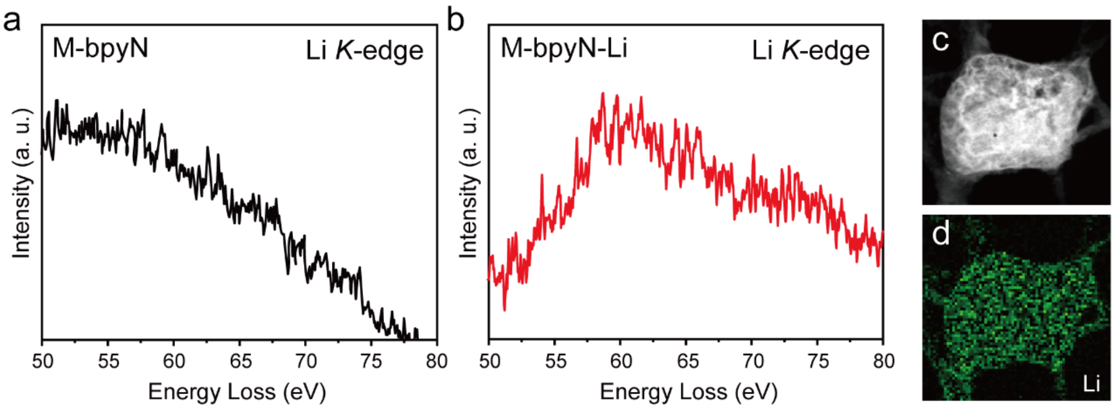

Figure S8. Comparison of EELS spectra of M-bpyN a) before and b) after Li deposition. C and d) Elemental mapping analysis of M-bpyN after Li deposition.

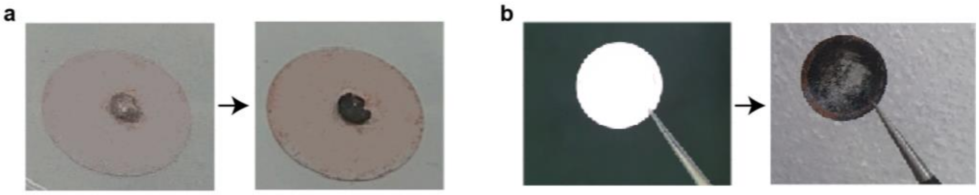

Figure S9. Images of Li metal on M-bpyN before and after various chemical activations, a) heat treatment, and b) charge–discharge cycling.

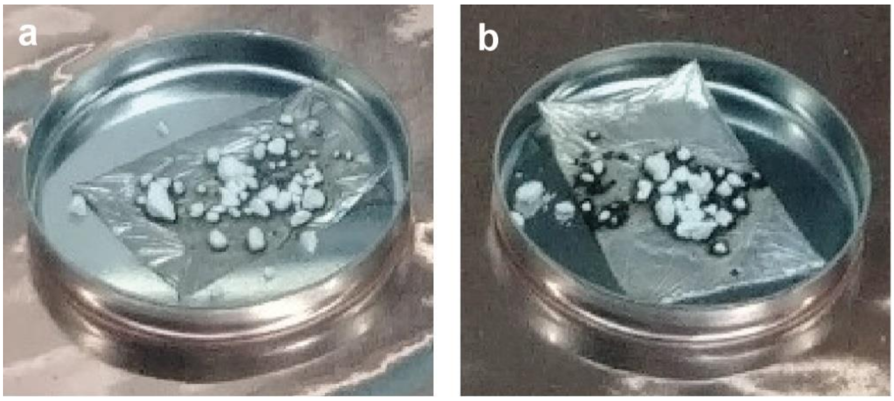

Figure S10. Color change of lithium foils through activation with a) bi-phenyl linker and b) bi-pyridine linker during heat treatment.

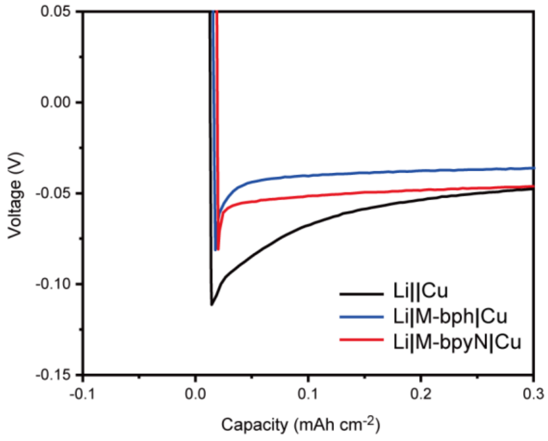

Figure S11. Comparison of nucleation overpotential at a current density of 1.0 mA cm^{-2}.

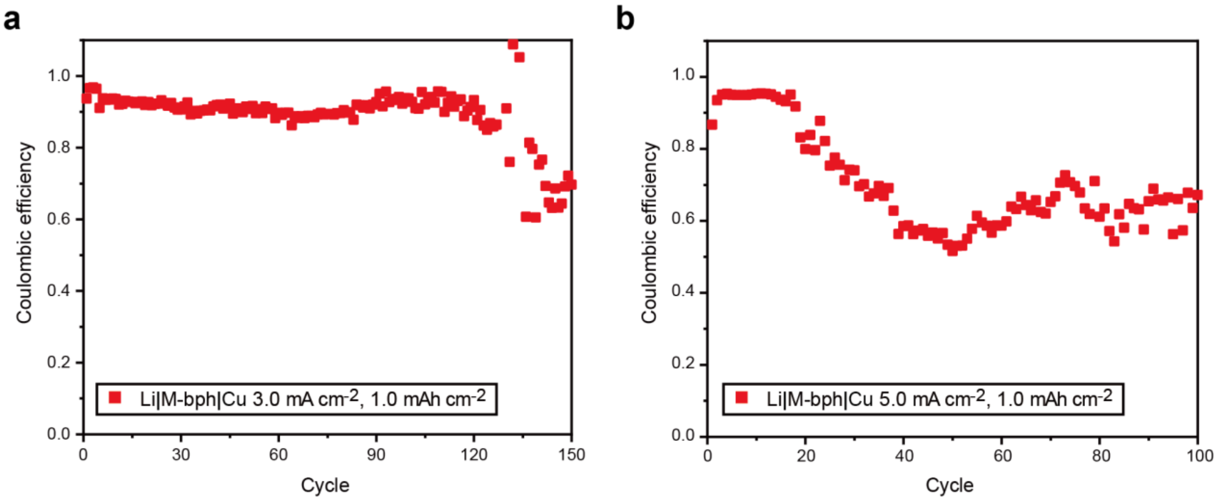

Figure S12. Cycle performances of the Li|M-bph|Cu configuration at high rate capabilities of a) 3.0 and b) 5.0 mA cm^{-2}, with an areal capacity of 1.0 mAh cm^{-2}.

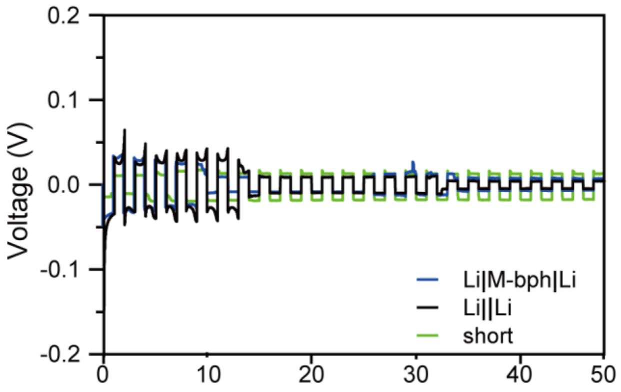

Figure S13. Voltage hysteresis comparison of the symmetric-cell configuration at various conditions.

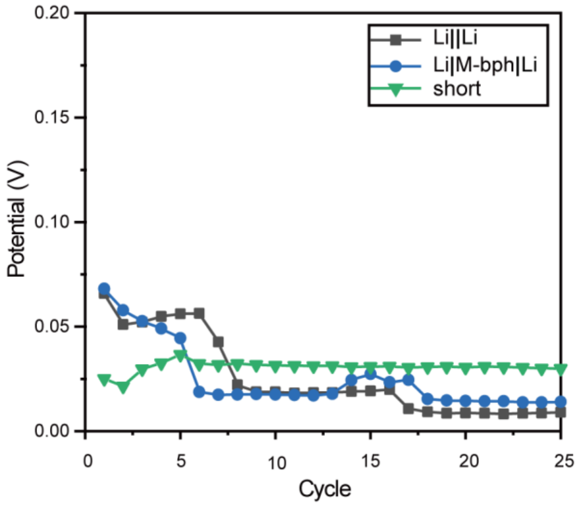

Figure S14. Overpotential comparison of the symmetric-cell configuration at various conditions. The overpotential of Li||Li and Li|M-bph|Li is decreased to that of the short cell configuration.

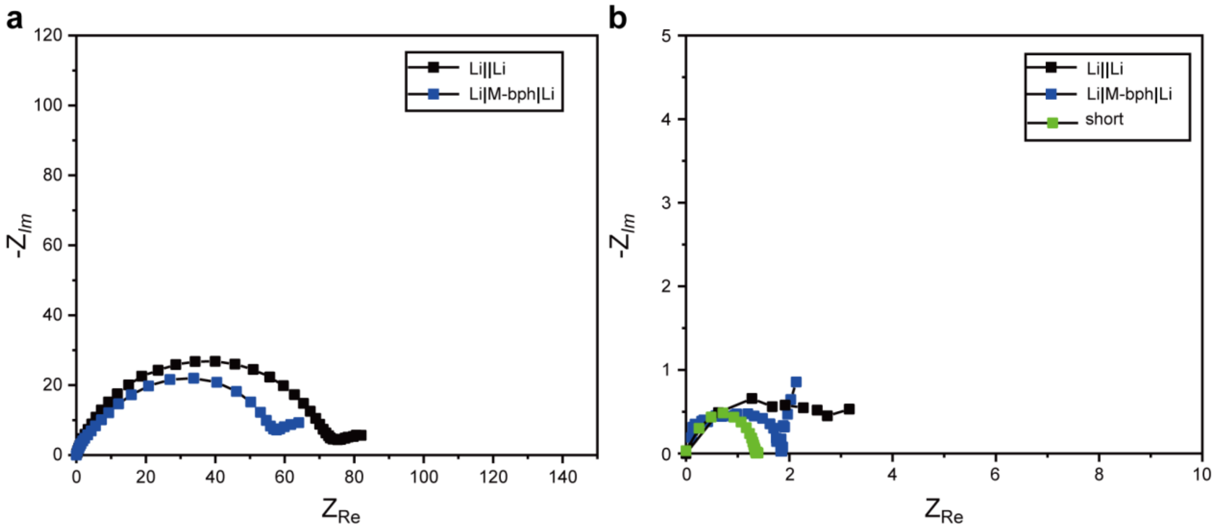

Figure S15. Electrochemical impedance spectroscopy (EIS) analyses of the symmetric-cell configuration. EIS spectra of (a) the as-assembled Li||Li, and Li|M-bph|Li symmetric-cell configurations and (b) comparison of EIS curves of Li||Li, and Li|M-bph|Li configurations with a short cell after undergoing cycling at 50 h.

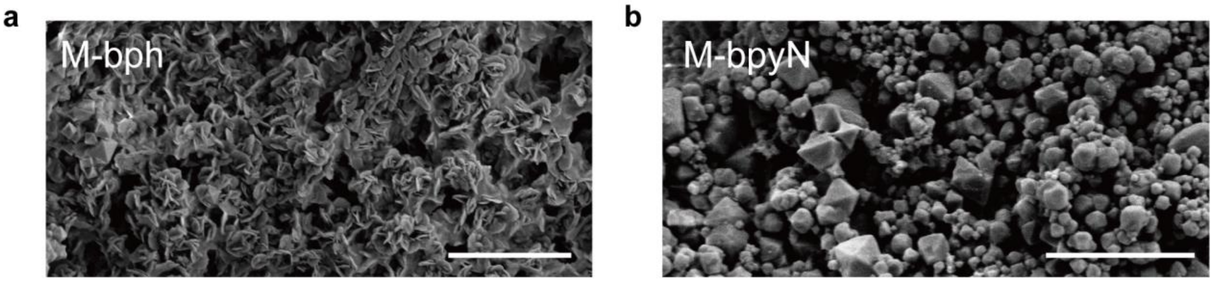

Figure S16. Morphology of a) M-bph and b) M-bpyN layers after repeated charge–discharge cycle in symmetric configuration. The scale bar is 2 μm.

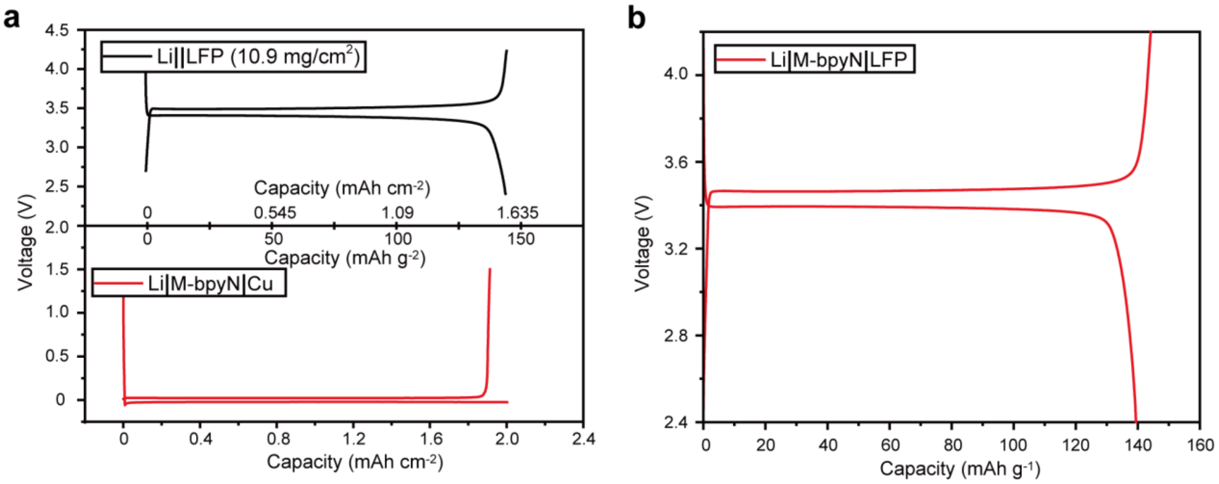

Figure S17. Electrochemical performance of the Li|M-bpyN|LFP configuration. a) Voltage profiles of positive and negative electrodes. b) Voltage profile of the Li|M-bpyN|LFP configuration during the first cycle at 0.1 C.

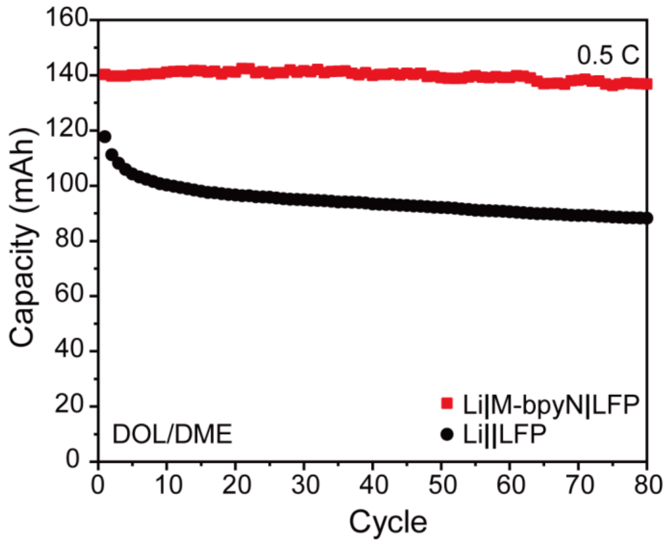

Figure S18. Cycle performance of Li||LFP and Li|M-bpyN|LFP using dioxalne (DOL)/ dimethoxyethane (DME) electrolytes at 0.5C.
